# The impact of gut microbiota on autoimmune thyroiditis and relationship with pregnancy outcomes: a review

**DOI:** 10.3389/fcimb.2024.1361660

**Published:** 2024-03-05

**Authors:** Yu Song, Yu Bai, Cong Liu, Xiaodan Zhai, Le Zhang

**Affiliations:** Department of Endocrinology, Shengjing Hospital of China Medical University, Shenyang, Liaoning, China

**Keywords:** autoimmune thyroiditis, AITD, TPOAb, gut microbiota, pregnancy outcomes

## Abstract

Autoimmune thyroiditis (AITD) is a T-cell-mediated, organ- specific autoimmune disease caused by interactions between genetic and environmental factors. Patients with AITD show thyroid lymphocyte infiltration and an increase in the titer of thyroid autoimmune antibodies, thereby altering the integrity of thyroid follicle epithelial cells and dysregulating their metabolism and immune function, leading to a decrease in multi-tissue metabolic activity. Research has shown that patients with AITD have a significantly higher risk of adverse pregnancy outcomes, such as infertility and miscarriage. Levothyroxine(LT_4_) treatment can improve the pregnancy outcomes of normal pregnant women with thyroid peroxidase antibodies(TPOAb) positivity, but it is not effective for invitro fertilization embryo transfer (IVF-ET) in women with normal thyroid function and positive TPOAb. Other factors may also influence pregnancy outcomes of patients with AITD. Recent studies have revealed that the gut microbiota participates in the occurrence and development of AITD by influencing the gut-thyroid axis. The bacterial abundance and diversity of patients with Hashimoto thyroiditis (HT) were significantly reduced, and the relative abundances of *Bacteroides*, *fecal Bacillus*, *Prevotella*, and *Lactobacillus* also decreased. The confirmation of whether adjusting the composition of the gut microbiota can improve pregnancy outcomes in patients with AITD is still pending. This article reviews the characteristics of the gut microbiota in patients with AITD and the current research on its impact in pregnancy.

## 1 Background

Autoimmune thyroiditis (AITD) is a T-cell-mediated, organ-specific autoimmune disease that mainly manifests as Hashimoto’s thyroiditis (HT) and Graves’ disease (GD) (Antonelli et al., 2015). The incidence rate of AITD is approximately 5%, and is more common in women of childbearing age (Lee et al., 2015). It is accompanied by lymphocyte infiltration and elevated titers of thyroid autoimmune antibodies, such as thyroid peroxidase antibodies (TPOAb) and thyroid globulin antibodies (TgAb) (Fröhlich and Wahl, 2017), which are associated with varying degrees of hypothyroidism (Caturegli et al., 2014). Infiltrating lymphocytes can directly produce cytotoxicity in thyroid follicular cells or may indirectly affect their vitality and function through cytokines; this alters cell integrity and dysregulates their metabolism and immune function, leading to thyroid gland enlargement, gland fibrosis, decreased thyroid hormone (TH) levels, and ultimately reduced metabolic activity in multiple tissues (Ajjan and Weetman, 2015; Mori et al., 2012). It can cause a decrease in cardiovascular contractility and intestinal activity, coronary artery disease, hyperlipidemia, infertility, and neurosensory and musculoskeletal changes (Chaker et al., 2017).Therefore, it is crucial to reduce the incidence of AITD.

The etiology of AITD remains unclear. Epidemiological studies have shown that AITD is caused by interactions between genetic and environmental factors (Taylor et al., 2018). Genetic susceptibility plays a crucial role in autoimmune disorders, and immune modification genes (such as human leukocyte antigen classes I and II) and sites related to cytotoxic T lymphocyte-associated protein 4 (CTLA-4) may be involved in the autoimmune process. The interactions between these gene loci and environmental factors may affect the phenotype and severity of HT (Ajjan and Weetman, 2015). Environmental factors that may trigger the development of AITD include excessive iodine intake; deficiencies in selenium, iron, zinc, and vitamin D; intake of gluten (Liontiris and Mazokopakis, 2017), and alcohol; excessive stress; pregnancy; and the use of interferon, key immune modulators, such as iprimumab and alenzumab (Topliss, 2016).However, a study has found that smoking can reduce the risk of AITD (Effraimidis and Wiersinga, 2014). Recently, extensive research has indicated that the gut microbiota may play an important role in triggering AITD (Köhling et al., 2017), thus providing new ideas for treating AITD.

## 2 The correlation between AITD and gut microbiota

### 2.1 Gut microbiota

Gut microbiota is a general term for the microorganisms that parasitize the human intestine. It comprises bacteria, fungi, viruses, and archaea, with bacteria accounting for the majority. There are approximately 2000 species of gut microbiota, and more than 100 species have been identified by phylum classification. The main phylum categories include Firmicutes, Bacteroidetes, Proteobacteria, Actinobacteria, and Verrucomycetes (Hardin et al., 2019). Among them, Firmicutes and Bacteroidetes account for > 90% of gut microbiota. The Firmicutes phylum has the highest number of bacteria, consisting of over 200 genera, including Lactobacillus, Mycoplasma, Bacillus, and Clostridium. The phylum Bacteroidetes includes more than 20 genera (Benson et al., 2010).

The gut microbiota undergoes corresponding changes owing to factors such as host genetics, diet, and environment, which can promote the growth of pathogenic bacteria (Kashtanova et al., 2016). Dysfunction of the gut microbiota not only causes a variety of gastrointestinal diseases, such as diarrhea, constipation, and enteritis, but can also induce chronic diseases, such as obesity, cardiovascular disease, diabetes, and metabolic syndrome (Marchesi et al., 2016; Cho and Blaser, 2012). Recent research has also shown that the intestinal flora and its metabolites may play a key role in the regulation of the immune system response and the development of autoimmune diseases, such as rheumatoid arthritis (RA) (Sun et al., 2019),multiple sclerosis (MS) (Cantoni et al., 2022), systemic lupus erythematosus(SLE) (Luo et al., 2018), type 1 diabetes(T1D) (Knip and Honkanen, 2017), and HT (Belvoncikova et al., 2022). The abundance of *Prevotella* in the feces of RA patients is higher (Alpizar-Rodriguez et al., 2019), and the genera *Faecalibacterium* and *Bacteroides* are reduced (Maeda and Takeda, 2019). *Prevotella* and *Pseudomonas* typically shows a decrease in the feces of patients with MS (Miyake et al., 2015), while the *Akkermansia muciniphila* typically increase (Ventura et al., 2019). Gut microbial diversity is significantly lower in patients with SLE with active disease than in non-SLE controls (Luo et al., 2018). In SLE patients, the relative abundance of *Firmicutes* decreased compared to the non-SLE controls, while *Bacteroidetes* increased (Hevia et al., 2014). A study conducted by Knip et al., to explore the relationship between gut microbiota and T1D, showed that children with positive islet-autoantibodies had a higher Bacteroidetes/Firmicutes ratio and lower Shannon diversity in the gut microbiota (Knip and Honkanen, 2017).

### 2.2 Characteristics of gut microbiota in patients with AITD

As shown in **Table 1**, some studies have proposed compositional modifications and bacterial ecological imbalances arise in the gut microbiota of patients with AITD, indicating that specific bacterial overgrowth and its impact on the gut-thyroid axis may play key roles in the occurrence and progression of AITD (Knezevic et al., 2020). This cross-sectional study compared 45 patients with HT of normal thyroid function (HTN), 18 patients with HT of hypothyroid status (HTH), and 34 healthy controls (CON). The bacterial abundance and diversity in patients with HTN and HTH were significantly lower than those in the healthy group, and patients with HTH showed the lowest intestinal microbial abundance (Liu et al., 2020a).

**Table 1:** Characteristics of gut microbiota in patients with autoimmune thyroid disease.

Number	Country	Reference	Period	Size				Result	
				GD	HTN	HTH	CON	Bacterial abundance	Specific differences in the microbiota
1	China	Liu et al., 2020a	2020	–	45	18	34	The microbial richness of gut microbiota in HT patients was significantly lower than in the control group. HT patients with hypothyroidism exhibited the least gut microbial abundance.	HT patients with euthyroidism have more Lachnospiraceae_incertae_sedis, Lactonifactor, Alistipes, and Subdoligranulum, while HT with hypothyroidism have more Phascolarctobacterium.Phascolarctobacterium may be involved in the progression of HT in humans.
2	China	Zhao et al., 2018	2018	–	28	–	16	Similar levels of bacterial richness and diversity were found in the gut microbiota of HT patients and healthy controls.	The abundance levels of Blautia, Roseburia, Ruminococcus_torques_group, Romboutsia, Dorea, Fusicatenibacter, and Eubacterium_hallii_group genera were increased in HT patients, whereas the abundance levels of Fecalibacterium, Bacteroides, Prevotella_9, and Lachnoclostridium genera were decreased.
3	China	Zhao et al., 2022	2022	27	–	16	3	The gut microbiota abundance and diversity in the GD and HT groups were similar to those in the healthy groups, but the overall structure was different.	Compared to Graves’ disease patients, HT patients are more abundant in Firmicutes, and have less Bacteroides, more Proteobacteria and Actinobacteria than the normal control group.Bacillus, Blautia, and Ornithinimicrobium can be used as potential markers to distinguish GD and HT patients from the healthy people.
4	China	Ishaq et al., 2017	2017	–	–	29	12	The richness and diversity of bacterial community were calculated at the 97% similarity level.The diversity elevation indicates a clear gut microbial overgrowth in patients group in contrast to healthy control.	The abundance of Prevotella_9 and Dialister declines in HT group,while Escherichia-Shigella and Parasutterella elevate.At the species leve,it also showed an increased abundance of E. coli in HT.

Sequencing analysis by Zhao et al. identified specific differences in the microbiota. The feces of patients with HT showed an increase in Firmicutes and Actinobacteria levels, whereas Bacteroides and Proteobacteria decreased. The ratio of Firmicutes to Bacteroides was significantly increased, and patients with HTN had a higher abundance and diversity of gut microbiota than the CON group (Zhao et al., 2018). A recent study found that compared to patients with Graves’ disease, patients with HT had more abundant Firmicutes, fewer Bacteroidetes, and more Proteobacteria and Actinobacteria levels than the normal control group (Zhao et al., 2022). Ishaq et al. also proposed that the relative abundance of Proteobacteria in the feces of patients with HT was significantly increased, whereas the relative abundance of Firmicutes and Bacteroidetes was decreased (Ishaq et al., 2017). These three studies found that the HT group had high levels of Spirochaetaceae, Enterobacteriaceae, Alcaligenaceae, Trichocomaceae, Erythrobacteraceae, and Bacteroidaceae. In contrast, the levels of *Prevotella*, *Ruminococcus*, and *Vibrio* were decreased in the HT group (Zhao et al., 2018; Zhao et al., 2022; Ishaq et al., 2017).

At the genus level, the relative abundances of *Bacteroides*, fecal *Bacillus*, *Prevotella*, and *Lactobacillus* in the fecal samples of patients with HT decreased, while the relative abundances of *Blautia*, *Ruminococcus*, *Rose*, *Clostridium*, *Longbuti*, *Dorea*, and *Eubacterium* increased significantly (Zhao et al., 2018). Studies have also suggested a decrease in Prevotella levels in the feces of patients with HT (Ishaq et al., 2017). A meta-analysis showed that the abundance of Firmicutes, Bifidobacteria, and Lactobacillus in patients with AITD was lower than that in healthy controls; patients with HT having slightly higher levels of Bacteroides than in other bacteria. These taxa are associated with clinical indicators, such as an altered host metabolism or TPOAb and TgAb positivity in the host (Gong et al., 2021). A cross-sectional study of 22 patients with HT and 11 healthy individuals conducted by Zhao et al. showed that 18 genera in the microbiota of patients with HT were positively correlated with TPOAb or TgAb, whereas six genera were negatively correlated. In addition, the Heterobacteria genus is positively correlated with free thyroxine, Clostridium genus is negatively correlated with free thyroxine, and Pleurotus genus is negatively correlated with serum thyrotropin (TSH) (Zhao et al., 2018).

### 2.3 The mechanism of gut microbiota affecting the development of AITD

**Figure 1:**
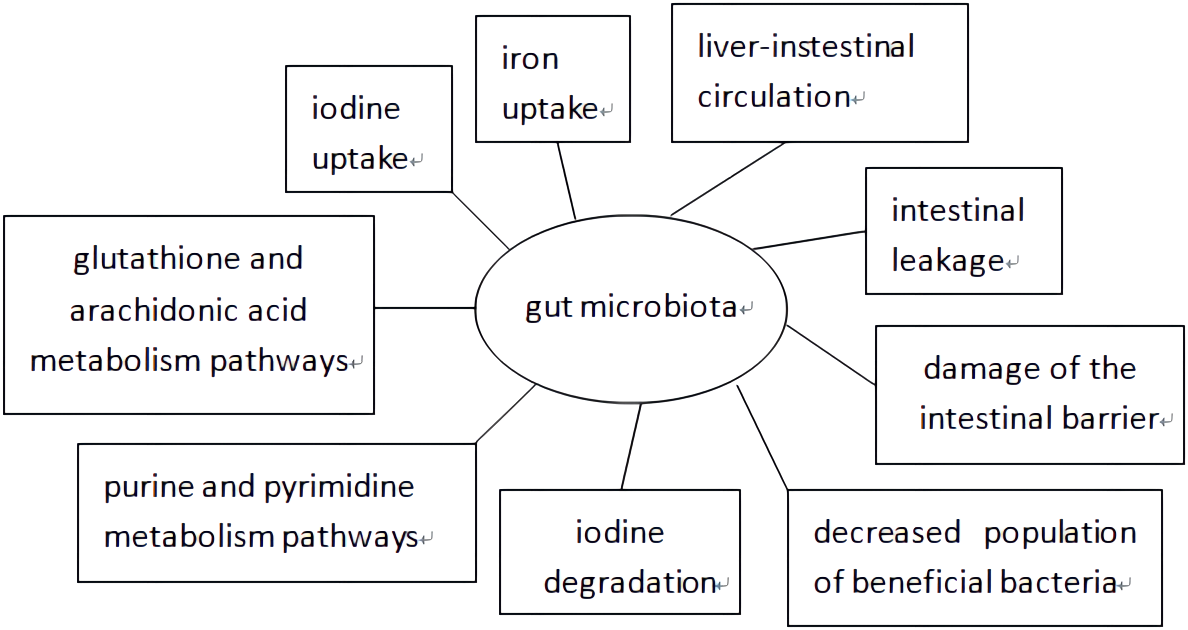
The possible mechanism of gut microbiota affecting the development of AITD.

As shown in **Figure 1**, extensive research has been conducted on the mechanism by which the gut microbiota affects AITD development. Minerals such as selenium, iron, and zinc have a significant impact on the interactions between the host and gut microbiota (Knezevic et al., 2020), which affect TH levels by regulating iodine uptake, degradation, and hepatic-intestinal circulation (Fröhlich and Wahl, 2019). The gut microbiota produces its own antigens through protein post-translational modifications, activates Toll-like receptor 4 induced by lipopolysaccharide (LPS), induces T helper cell translocation from type 1 (Th1) to type 2 (Th2), reduces the integrity of intercellular connections, and promotes AITD development through intestinal leakage (Lerner et al., 2017). Some scholars also believe that changes in gut microbiota occur through post-translational modifications of luminal proteins, the transition of the intestinal mucosa to a pro-inflammatory environment, intestinal ecological imbalances leading to damage of the intestinal barrier, antigen entry into the circulation, activation of the immune system antibodies in the circulation, which react with bacterial antigens to enhance inflammatory body activations in the thyroid gland, and excessive bacterial growth that participates in the development of autoimmune thyroiditis (Mu et al., 2017; Cayres et al., 2021; Tomasello et al., 2015). Another theory suggests that a decreased population of beneficial bacteria such as Lactobacillus and Bifidobacterium is related to the development of AITD. Lactobacillus has been proven to protect TH17 cells and support their barrier integrity by secreting IL-22 and IL-17. The Th17/Treg imbalance may cause inflammatory disorders, indicating that Lactobacillus participates in the immune system balance. Bifidobacterium and Lactobacillus exhibit anti-inflammatory effects and protect the body from pathogens. Moreover, increased Bacteroides fragilis may account for the upregulation of IL-18, IL-1β, and caspase-1, promoting an inflammatory response (Kiseleva et al., 2011). It has been proposed that bacterial strains participate in the development of HT by influencing glutathione and arachidonic acid metabolism, and purine and pyrimidine metabolism pathways; however, further validation is still needed (Zhao et al., 2022).

## 3 The impact of AITD on pregnancy

Numerous studies have shown that AITD increases the risk of adverse pregnancy outcomes. Women may experience changes in hormone levels and metabolic needs during pregnancy, such as an increase demand for THs to meet the needs of fetal growth and brain development. Therefore, thyroid diseases are frequently observed during pregnancy (Krassas et al., 2010). Thyroid dysfunction during pregnancy can include overt hypothyroidism (OH) and subclinical hypothyroidism (SCH). The relative incidence rates of OH and SCH are approximately 0.3–1.0% and 4.0–17.8%, respectively (Shan and Wang, 2022). AITD is the main cause of hypothyroidism in pregnant women, with an average incidence of 7.8% (Krassas et al., 2015).

### 3.1 AITD and infertility

The incidence of infertility in women with AITD is high, with a prevalence of 52.3% in patients with GD and 47.0% in patients with HT (Quintino-Moro et al., 2014). In a prospective study, 438 women with infertility and 100 healthy women in postpartum were compared, and it was found that the prevalence of TPOAb positivity was significantly higher among women with infertility factors than those of the healthy group (Poppe et al., 2002).

### 3.2 AITD and miscarriage

AITD is associated with recurrent miscarriage (RM). Some scholars believe that autoimmunity in women with AITD affects embryo implantation by inducing endometrial receptive defects (Kim et al., 2011; Liu et al., 2020b; Wu et al., 2019), leading to an increase in fetal miscarriages. Some scholars also believe that, in women affected by thyroid autoimmunity, the thyroid may have insufficient TH release in the early stages of pregnancy, and their increased miscarriage rate may be due to TH deficiency rather than a systemic overreaction of the immune system (Abalovich et al., 2007). The local effects of TH on female reproductive organs and embryos during embryo implantation are crucial for successful pregnancies (Stavreus Evers, 2012).

A prospective cohort study conducted in women with infertility found that the median serum TSH levels were significantly higher in TPOAb- and TgAb-positive women than in women without AITD (Unuane et al., 2013). The TSH level is a sensitive indicator of thyroid function during pregnancy (Tortosa, 2011). The upper limit of the normal value of TSH in early pregnancy should be 4.0 mU/L, and 2.5 mU/L≤ TSH< 4.0 mU/L is called the normal high value of TSH. Women with positive thyroid antibodies or those undergoing assisted reproduction require levothyroxine (LT_4_) (Shan and Wang, 2022). Therefore, some scholars used LT_4_ intervention as adjuvant therapy in 227 women with AITD who suffered from RM and it was found that low-dose LT_4_ treatment can, to some extent, prevent miscarriage (Dal Lago et al., 2021). Another study also showed that administering LT_4_ treatment to pregnant women with a history of hypothyroidism and TPOAb-positivity can improve their live birth rates and reduce miscarriages (Leng et al., 2022). However, some studies have found that LT_4_ treatment did not increase live birth rates in women with RM, normal thyroid function, and positive TPOAb (van Dijk et al., 2022). Hong et al. also confirmed that LT_4_ treatment did not reduce miscarriage rates or increase live birth rates in women undergoing *in vitro* fertilization embryo transfer (IVF-ET) with intact thyroid function and positive TPOAb (Wang et al., 2017). The use of glucocorticoids and aspirin as adjunctive therapies in euthyroid women with AITD undergoing IVF-ET may not improve pregnancy or live birth rates either (Zhou et al., 2022).

### 3.3 AITD and other adverse pregnancy outcomes

After analyzing 35 studies, we found that TPOAb-positive women had a higher risk of premature birth than TPOAb-negative women. The relationship between TPOAb positivity and premature birth appears to be related to TSH concentration. TPOAb-positive women with TSH concentrations higher than 4.0 mU/L have a higher risk of premature birth (Korevaar et al., 2019). Tang et al. found that with an increase in TPOAb and TgAb (in early and mid-pregnancy), the maternal risk of gestational diabetes mellitus (GDM) significantly increased. Therefore, the presence of thyroid antibodies can predict postpartum glucose abnormalities in individuals with GDM (Tang et al., 2021). Some studies have evaluated the impact of LT_4_ on the risk of miscarriage, premature birth, preeclampsia, placental abruption, birth weight, gestational age at delivery, and neonatal admission rate in TPOAb-positive pregnant women with normal thyroid function; nevertheless, no significant differences between the LT_4_ administrated and control groups were found. However, there has been a downward trend in premature births and miscarriages.

## 4 Gut microbiota and pregnancy

In recent years, increasing evidence has shown that sex hormones can affect the structure of gut microbiota, and sex hormones act through steroid receptors directly regulate the metabolism of bacteria (Yoon and Kim, 2021). Autonomous diseases are typically more prevalent in women than in men (Quintero et al., 2012). A role for gut microbiota in the sex bias in autoimmunity has been revealed by different studies in animal models. This bias is at least partially mediated by the microbial metabolism of sex hormones (Ortona et al., 2016). Pregnancy is a special period for women, as the body undergoes various physiological changes, which provides the fetus with the best growth and development conditions (Costantine, 2014).Changes of hormones in pregnancy can alter the gut microbiota structure of pregnant women (Koren et al., 2012). As pregnancy progresses, there is a significant enrichment of *Neisseria, Brautia, Collins*, and *Bifidobacterium* genera. The increase in relative abundance of *Bifidobacterium* is highly likely mediated by progesterone (Nuriel-Ohayon et al., 2019). Throughout pregnancy, significant changes occur in the gut microbiota of mothers, which subsequently affect the gut microbiota of infants. Changes in microbiome composition occur between the first and third trimesters of pregnancy (Gorczyca et al., 2022). Scholars transplanted fecal microbiota from the first and third trimesters of pregnancy into sterile mice. Compared with mice transplanted with the first trimester of pregnancy microbiota, mice transplanted with the third trimester of microbiota showed significant weight gain, insulin resistance, and greater inflammatory response (Koren et al., 2012). Akkermansia, Bifidobacteria, and Firmicutes populations increase, which is related to an increase in energy storage requirements. Proteobacteria and Actinobacteria levels increase, owing to their pro-inflammatory properties, and have protective effects on both mothers and fetuses (Rodríguez et al., 2015). The mechanism of these changes involves the regulation of the brain and intestinal axes by production of maternal estrogen and progesterone, as well as immune activation of the intestinal mucosa (Mulak et al., 2014; Stanislawski et al., 2017).

As shown in **Table 2**, many studies have demonstrated that the gut microbiota is associated with many diseases during pregnancy. A study conducted among 100 women showed that 26 pregnant women with preeclampsia had a significantly lower abundance of *Prevotella*, *Porphyromonas*, *Varibaculum*, and *Lactobacillus* than pregnant women without this complication (Huang et al., 2021). Liu also reported significant structural changes in the gut microbiota of patients with preeclampsia. In these patients, there was an overall increase in the pathogenic bacteria *Clostridium perfringens* and *Bulleidia moorei*, but a reduction in the probiotic bacteria *Coprococcus catus* (Liu et al., 2017). Fetal growth restriction (FGR) is a common obstetric complication and also known as intrauterine growth restriction (IUGR) (Sharma et al., 2016). By 16S rDNA amplicon sequencing of samples, collected from pregnant women in the FGR and control groups, it was revealed that the genera *Bacteroides*, *Faecalibacterium*, and *Lachnospira* were highly abundant in the FGR group (Tu et al., 2022). GDM is one of the most common metabolic complications of pregnancy and its prevalence has significantly increased over the last few years (Filardi et al., 2019). Cortez et al. found an increase in Firmicutes and a decrease in Bacteroides levels in patients with GDM, as well as an increase in *Firmicutes*/*Bacteroides* (F/B) ratio in late pregnancy (Cortez et al., 2019). The increase in the F/B ratio is associated with low-grade inflammation, insulin resistance, and obesity (Pascale et al., 2019). Sililas et al. also found that the F/B ratio in the third trimester of pregnancy was higher in patients with GDM than in those of the control group (Sililas et al., 2021). Specific shifts in microbial composition were also associated with maternal factors such as BMI, weight, and weight gain during pregnancy. A higher number of *Bifidobacterium* organisms and lower levels of *Staphylococcus* may protect the mother from developing excess weight (Collado et al., 2008; Santacruz et al., 2010). A study found that overweight participants had significantly higher fecal concentrations of the genus *Bacteroides* and a lower F/B ratio (Schwiertz et al., 2010).

**Table 2:** Characteristics of gut microbiota in pregnant women with other diseases.

Number	Country	Reference	Period	Size		Result
				Case group	Control group	
1	Norway	Stanislawski et al., 2017	2017	116	–	The most important taxa among women with excess gestational weight gain (GWG) included *Methanobrevibacter, Bifidobacterium*, and *Bacteroides*, as well as seven OTUs of the order Clostridiales. There were three OTUs,include Blautia, SMB53, Methanobrevibacter, that were significantly higher among women with excess GWG.
2	China	Huang et al., 2021	2021	51	49	Pregnant women with preeclampsia had significantly lower abundance of Prevotella, Porphyromonas, Varibaculum, and Lactobacillus compared to those without this complication. The abundance of *Prevotella*, *Porphyromonas, Lactobacillus, Mobiluncus, Campylobacter* and *Peptostreptococcus* were decreased significantly in the pregnant women with abnormal placental growth
3	China	Liu et al., 2017	2016	26	74	In preeclampsia patients, there was an overall increase in pathogenic bacteria, Clostridium perfringens and Bulleidia moorei,but a reduction in probiotic bacteria Coprococcus catus
4	China	Tu et al., 2022	2022	14	18	At phylum level, *Firmicutes* was more abundant in the Fetal growth restriction(FGR) group than in the control group. At genus level, *Bacteroides, Faecalibacterium, Lachnospira* (all belong to *Lachnospiraceae* family) were highly abundant in the FGR group as compared to the control group.
5	Brazil	Cortez et al., 2019	2019	26	42	The GDM patients presented a significantly higher abundance of the genera Bacteroides, Veillonella, Klebsiella, Escherichia-Shigella, Enterococcus, and Enterobacter.There is an increase in Firmicutes and a decrease in Bacteroides in GDM patients, as well as an increase in Firmicutes/Bacteroides (F/B ratio) in late pregnancy.
6	Thailand	Sililas et al., 2021	2021	49	39	There is a reduction in *Lactobacillales* from the time of GDM diagnosis to the time before delivery (≥37 weeks gestation). F/B ratio was found higher in GDM mother, when compared to their non-GDM counterparts, at the time before delivery. However, these alterations were not observed in meconium and the first feces of their newborn.
7	Finland	Collado et al., 2008	2008	18	36	Bacteroides and Staphylococcus were significantly higher in the overweight state than in normal-weight women. Mother’s weight and BMI before pregnancy correlated with higher concentrations of Bacteroides, Clostridium, and Staphylococcus. Microbial counts increased from the first to third trimester of pregnancy. High Bacteroides concentrations were associated with excessive weight gain over pregnancy.
8	Spain	Santacruz et al., 2010	2010	16	34	Reduced numbers of Bifidobacterium and Bacteroides and increased numbers of Staphylococcus, Enterobacteriaceae and Escherichia coli were detected in overweight compared with normal-weight pregnant women. E. coli numbers were higher in women with excessive weight gain than in women with normal weight gain during pregnancy, while Bifidobacterium and Akkermansia muciniphila showed an opposite trend.

## 5 Summary

AITD increases the risk of infertility, miscarriage, and other adverse pregnancy and neonatal outcomes. The use of LT_4_ intervention can reduce adverse outcomes in patients with normally high TSH levels. However, it is not effective in euthyroid patients with AITD who undergo IVF-ET assisted pregnancy. It is not clear whether other factors affect adverse pregnancy outcomes in patients with AITD (van Dijk et al., 2022). Therefore, a new interventional approach is required to reduce adverse outcomes. Some researchers have found differences in the composition of the gut microbiota between patients with AITD and the normal population. Specific bacterial overgrowth and its impact on the gut-thyroid axis may promote thyroid antibody production. Currently, little research has explored the relationship between specific differences in gut microbiota composition in patients with AITD, and especially of those who are pregnant. It is unclear how the gut microbiota contributes to adverse pregnancy outcomes in TPOAb-positive women. Whether it is possible to improve the pregnancy outcomes of patients with AITD by regulating the composition of the gut microbiota still needs to be confirmed.

## Author contributions

YS: Writing – original draft, Writing – review & editing. YB: Writing – review & editing. CL: Writing – review & editing. XZ: Writing – review & editing. LZ: Writing – review & editing.

## References

[B1] Abalovich M. Amino N. Barbour L. A. Cobin R. H. De Groot L. J. Glinoer D.. (2007). Management of thyroid dysfunction during pregnancy and postpartum: an Endocrine Society Clinical Practice Guideline. J. Clin. Endocrinol. Metab. 92, S1–47. doi: 10.1210/jc.2007-0141 17948378

[B2] Ajjan R. A. Weetman A. P. (2015). The pathogenesis of hashimoto’s thyroiditis: further developments in our understanding. Horm. Metab. Res. 47, 702–710. doi: 10.1055/s-0035-1548832 26361257

[B3] Alpizar-Rodriguez D. Lesker T. R. Gronow A. Gilbert B. Raemy E. Lamacchia C.. (2019). Prevotella copri in individuals at risk for rheumatoid arthritis. Ann. Rheum Dis. 78, 590–593. doi: 10.1136/annrheumdis-2018-214514 30760471

[B4] Antonelli A.. (2015). Autoimmune thyroid disorders. Autoimmun Rev. 14, 174–180. doi: 10.1016/j.autrev.2014.10.016 25461470

[B5] Belvoncikova P. Maronek M. Gardlik R. (2022). Gut dysbiosis and fecal microbiota transplantation in autoimmune diseases. Int. J. Mol. Sci. 23 (18), 10729. doi: 10.3390/ijms231810729 36142642 PMC9503867

[B6] Benson A. K. Kelly S. A. Legge R. Ma F. Low S. J. Kim J.. (2010). Individuality in gut microbiota composition is a complex polygenic trait shaped by multiple environmental and host genetic factors. Proc. Natl. Acad. Sci. U.S.A. 107, 18933–18938. doi: 10.1073/pnas.1007028107 20937875 PMC2973891

[B7] Cantoni C. Lin Q. Dorsett Y. Ghezzi L. Liu Z. Pan Y.. (2022). Alterations of host-gut microbiome interactions in multiple sclerosis. EBioMedicine 76, 103798. doi: 10.1016/j.ebiom.2021.103798 35094961 PMC8814376

[B8] Caturegli P. De Remigis A. Rose N. R. (2014). Hashimoto thyroiditis: clinical and diagnostic criteria. Autoimmun Rev. 13, 391–397. doi: 10.1016/j.autrev.2014.01.007 24434360

[B9] Cayres L. C. F. de Salis L. V. V. Rodrigues G. S. P. Lengert A. V. H. Biondi A. P. C. Sargentini L. D. B.. (2021). Detection of alterations in the gut microbiota and intestinal permeability in patients with hashimoto thyroiditis. Front. Immunol. 12, 579140. doi: 10.3389/fimmu.2021.579140 33746942 PMC7973118

[B10] Chaker L. Bianco A. C. Jonklaas J. Peeters R. P. (2017). Hypothyroidism. Lancet 390, 1550–1562. doi: 10.1016/S0140-6736(17)30703-1 28336049 PMC6619426

[B11] Cho I. Blaser M. J. (2012). The human microbiome: at the interface of health and disease. Nat. Rev. Genet. 13, 260–270. doi: 10.1038/nrg3182 22411464 PMC3418802

[B12] Collado M. C. Isolauri E. Laitinen K. Salminen S. (2008). Distinct composition of gut microbiota during pregnancy in overweight and normal-weight women. Am. J. Clin. Nutr. 88, 894–899. doi: 10.1093/ajcn/88.4.894 18842773

[B13] Cortez R. V. Taddei C. R. Sparvoli L. G. Ângelo A. G. S. Padilha M.. (2019). Microbiome and its relation to gestational diabetes. Endocrine 64, 254–264. doi: 10.1007/s12020-018-1813-z 30421135

[B14] Costantine M. M. (2014). Physiologic and pharmacokinetic changes in pregnancy. Front. Pharmacol. 5, 65. doi: 10.3389/fphar.2014.00065 24772083 PMC3982119

[B15] Dal Lago A. Galanti F. Miriello D. Marcoccia A. Massimiani M. Campagnolo L.. (2021). Positive impact of levothyroxine treatment on pregnancy outcome in euthyroid women with thyroid autoimmunity affected by recurrent miscarriage. J. Clin. Med. 10, 10 2105. doi: 10.3390/jcm10102105 PMC8153344 34068288

[B16] Effraimidis G. Wiersinga W. M. (2014). Mechanisms in endocrinology: autoimmune thyroid disease: old and new players. Eur. J. Endocrinol. 170, R241–R252. doi: 10.1530/EJE-14-0047 24609834

[B17] Filardi T. Panimolle F. Crescioli C. Lenzi A. Morano S. (2019). Gestational diabetes mellitus: the impact of carbohydrate quality in diet. Nutrients 11 (7), 1549. doi: 10.3390/nu11071549 31323991 PMC6683084

[B18] Fröhlich E. Wahl R. (2017). Thyroid autoimmunity: role of anti-thyroid antibodies in thyroid and extra-thyroidal diseases. Front. Immunol. 8, 521. doi: 10.3389/fimmu.2017.00521 28536577 PMC5422478

[B19] Fröhlich E. Wahl R. (2019). Microbiota and thyroid interaction in health and disease. Trends Endocrinol. Metab. 30, 479–490. doi: 10.1016/j.tem.2019.05.008 31257166

[B20] Gong B. Wang C. Meng F. Wang H. Song B. Yang Y.. (2021). Association between gut microbiota and autoimmune thyroid disease: A systematic review and meta-analysis. Front. Endocrinol. (Lausanne) 12, 774362. doi: 10.37766/inplasy2021.4.0135 34867823 PMC8635774

[B21] Gorczyca K. Obuchowska A. Kimber-Trojnar Ż. Wierzchowska-Opoka M. Leszczyńska-Gorzelak B. (2022). Changes in the gut microbiome and pathologies in pregnancy. Int. J. Environ. Res. Public Health 19. doi: 10.3390/ijerph19169961 PMC9408136 36011603

[B22] Hardin S. J. Singh M. Eyob W. Molnar J. C. Homme R. P. George A. K.. (2019). Diet-induced chronic syndrome, metabolically transformed trimethylamine-N-oxide, and the cardiovascular functions. Rev. Cardiovasc. Med. 20, 121–128. doi: 10.31083/j.rcm.2019.03.518 31601086

[B23] Hevia A. Milani C. López P. Cuervo A. Arboleya S. Duranti S.. (2014). Intestinal dysbiosis associated with systemic lupus erythematosus. mBio 5, e01548–e01514. doi: 10.1128/mBio.01548-14 25271284 PMC4196225

[B24] Huang L. Cai M. Li L. Zhang X. Xu Y. Xiao J.. (2021). Gut microbiota changes in preeclampsia, abnormal placental growth and healthy pregnant women. BMC Microbiol. 21, 265. doi: 10.1186/s12866-021-02327-7 34607559 PMC8489045

[B25] Ishaq H. M. Mohammad I. S. Guo H. Shahzad M. Hou Y. J. Ma C.. (2017). Molecular estimation of alteration in intestinal microbial composition in Hashimoto’s thyroiditis patients. BioMed. Pharmacother. 95, 865–874. doi: 10.1016/j.biopha.2017.08.101 28903182

[B26] Kashtanova D. A. Popenko A. S. Tkacheva O. N. Tyakht A. B. Alexeev D. G. Boytsov S. A. (2016). Association between the gut microbiota and diet: Fetal life, early childhood, and further life. Nutrition 32, 620–627. doi: 10.1016/j.nut.2015.12.037 26946974

[B27] Kim N. Y. Cho H. J. Kim H. Y. Yang K. M. Ahn H. K. Thornton S.. (2011). Thyroid autoimmunity and its association with cellular and humoral immunity in women with reproductive failures. Am. J. Reprod. Immunol. 65, 78–87. doi: 10.1111/aji.2010.65.issue-1 20712806

[B28] Kiseleva E. P. Mikhailopulo K. I. Sviridov O. V. Novik G. I. Knirel Y. A. Szwajcer Dey E. (2011). The role of components of Bifidobacterium and Lactobacillus in pathogenesis and serologic diagnosis of autoimmune thyroid diseases. Benef Microbes 2, 139–154. doi: 10.3920/BM2010.0011 21831795

[B29] Knezevic J. Starchl C. Tmava Berisha A. Amrein K. (2020). Thyroid-gut-axis: how does the microbiota influence thyroid function? Nutrients 12 (2), 1769. doi: 10.3390/nu12061769 32545596 PMC7353203

[B30] Knip M. Honkanen J. (2017). Modulation of type 1 diabetes risk by the intestinal microbiome. Curr. Diabetes Rep. 17, 105. doi: 10.1007/s11892-017-0933-9 28942491

[B31] Köhling H. L. Plummer S. F. Marchesi J. R. Davidge K. S. Ludgate M. (2017). The microbiota and autoimmunity: Their role in thyroid autoimmune diseases. Clin. Immunol. 183, 63–74. doi: 10.1016/j.clim.2017.07.001 28689782

[B32] Koren O. Goodrich J. K. Cullender T. C. Spor A. Laitinen K. Bäckhed H. K.. (2012). Host remodeling of the gut microbiome and metabolic changes during pregnancy. Cell 150, 470–480. doi: 10.1016/j.cell.2012.07.008 22863002 PMC3505857

[B33] Korevaar T. I. M. Derakhshan A. Taylor P. N. Meima M. Chen L. Bliddal S.. (2019). Association of thyroid function test abnormalities and thyroid autoimmunity with preterm birth: A systematic review and meta-analysis. Jama 322, 632–641. doi: 10.1001/jama.2019.10931 31429897 PMC6704759

[B34] Krassas G. Karras S. N. Pontikides N. (2015). Thyroid diseases during pregnancy: a number of important issues. Hormones (Athens) 14, 59–69. doi: 10.1007/BF03401381 25885104

[B35] Krassas G. E. Poppe K. Glinoer D. (2010). Thyroid function and human reproductive health. Endocr. Rev. 31, 702–755. doi: 10.1210/er.2009-0041 20573783

[B36] Lee H. J. Li C. W. Hammerstad S. S. Stefan M. Tomer Y. (2015). Immunogenetics of autoimmune thyroid diseases: A comprehensive review. J. Autoimmun 64, 82–90. doi: 10.1016/j.jaut.2015.07.009 26235382 PMC4628844

[B37] Leng T. Li X. Zhang H. (2022). Levothyroxine treatment for subclinical hypothyroidism improves the rate of live births in pregnant women with recurrent pregnancy loss: a randomized clinical trial. Gynecol Endocrinol. 38, 488–494. doi: 10.1080/09513590.2022.2063831 35426326

[B38] Lerner A. Jeremias P. Matthias T. (2017). Gut-thyroid axis and celiac disease. Endocr. Connect 6, R52–r58. doi: 10.1530/EC-17-0021 28381563 PMC5435852

[B39] Liontiris M. I. Mazokopakis E. E. (2017). A concise review of Hashimoto thyroiditis (HT) and the importance of iodine, selenium, vitamin D and gluten on the autoimmunity and dietary management of HT patients.Points that need more investigation. Hell J. Nucl. Med. 20, 51–56. doi: 10.1967/s002449910507 28315909

[B40] Liu J. Yang H. Yin Z. Jiang X. Zhong H. Qiu D.. (2017). Remodeling of the gut microbiota and structural shifts in Preeclampsia patients in South China. Eur. J. Clin. Microbiol. Infect. Dis. 36, 713–719. doi: 10.1007/s10096-016-2853-z 27988814

[B41] Liu S. An Y. Cao B. Sun R. Ke J. Zhao D. (2020a). The composition of gut microbiota in patients bearing hashimoto’s thyroiditis with euthyroidism and hypothyroidism. Int. J. Endocrinol. 2020 p, 5036959. doi: 10.1155/2020/5036959 PMC7673947 33224194

[B42] Liu S. Xu F. Wei H. Huang C. Chen X. Lian R.. (2020b). The correlation of thyroid autoimmunity and peripheral and uterine immune status in women with recurrent miscarriage. J. Reprod. Immunol. 139, 103118. doi: 10.1016/j.jri.2020.103118 32193011

[B43] Luo X. M. Edwards M. R. Mu Q. Yu Y. Vieson M. D. Reilly C. M.. (2018). Gut microbiota in human systemic lupus erythematosus and a mouse model of lupus. Appl. Environ. Microbiol. 84 (4), e02288-17. doi: 10.1128/AEM.02288-17 29196292 PMC5795066

[B44] Maeda Y. Takeda K. (2019). Host-microbiota interactions in rheumatoid arthritis. Exp. Mol. Med. 51, 1–6. doi: 10.1038/s12276-019-0283-6 PMC6906371 31827063

[B45] Marchesi J. R. Adams D. H. Fava F. Hermes G. D. Hirschfield G. M. Hold G.. (2016). The gut microbiota and host health: a new clinical frontier. Gut 65, 330–339. doi: 10.1136/gutjnl-2015-309990 26338727 PMC4752653

[B46] Miyake S. Kim S. Suda W. Oshima K. Nakamura M. Matsuoka T.. (2015). Dysbiosis in the gut microbiota of patients with multiple sclerosis, with a striking depletion of species belonging to clostridia XIVa and IV clusters. PloS One 10, e0137429. doi: 10.1371/journal.pone.0137429 26367776 PMC4569432

[B47] Mori K. Nakagawa Y. Ozaki H. (2012). Does the gut microbiota trigger Hashimoto’s thyroiditis? Discovery Med. 14, 321–326. 23200063

[B48] Mu Q. Kirby J. Reilly C. M. Luo X. M. (2017). Leaky gut as a danger signal for autoimmune diseases. Front. Immunol. 8, 598. doi: 10.3389/fimmu.2017.00598 28588585 PMC5440529

[B49] Mulak A. Taché Y. Larauche M. (2014). Sex hormones in the modulation of irritable bowel syndrome. World J. Gastroenterol. 20, 2433–2448. doi: 10.3748/wjg.v20.i10.2433 24627581 PMC3949254

[B50] Nuriel-Ohayon M. Neuman H. Ziv O. Belogolovski A. Barsheshet Y. Bloch N.. (2019). Progesterone increases bifidobacterium relative abundance during late pregnancy. Cell Rep. 27, 730–736.e3. doi: 10.1016/j.celrep.2019.03.075 30995472

[B51] Ortona E. Pierdominici M. Maselli A. Veroni C. Aloisi F. Shoenfeld Y. (2016). Sex-based differences in autoimmune diseases. Ann. Ist Super Sanita 52, 205–212. doi: 10.4415/ANN_16_02_12 27364395

[B52] Pascale A. Marchesi N. Govoni S. Coppola A. Gazzaruso C. (2019). The role of gut microbiota in obesity, diabetes mellitus, and effect of metformin: new insights into old diseases. Curr. Opin. Pharmacol. 49, 1–5. doi: 10.1016/j.coph.2019.03.011 31015106

[B53] Poppe K. Glinoer D. Van Steirteghem A. Tournaye H. Devroey P. Schiettecatte J.. (2002). Thyroid dysfunction and autoimmunity in infertile women. Thyroid 12, 997–1001. doi: 10.1089/105072502320908330 12490077

[B54] Quintero O. L. Amador-Patarroyo M. J. Montoya-Ortiz G. Rojas-Villarraga A. Anaya J. M. (2012). Autoimmune disease and gender: plausible mechanisms for the female predominance of autoimmunity. J. Autoimmun 38, J109–J119. doi: 10.1016/j.jaut.2011.10.003 22079680

[B55] Quintino-Moro A. Zantut-Wittmann D. E. Tambascia M. Machado Hda C. Fernandes A. (2014). High prevalence of infertility among women with graves’ Disease and hashimoto’s thyroiditis. Int. J. Endocrinol. 2014, 982705. doi: 10.1155/2014/982705 24678319 PMC3942334

[B56] Rodríguez J. M. Murphy K. Stanton C. Ross R. P. Kober O. I. Juge N.. (2015). The composition of the gut microbiota throughout life, with an emphasis on early life. Microb. Ecol. Health Dis. 26, 26050. doi: 10.3402/mehd.v26.26050 25651996 PMC4315782

[B57] Santacruz A. Collado M. C. García-Valdés L. Segura M. T. Martín-Lagos J. A. Anjos T.. (2010). Gut microbiota composition is associated with body weight, weight gain and biochemical parameters in pregnant women. Br. J. Nutr. 104, 83–92. doi: 10.1017/S0007114510000176 20205964

[B58] Schwiertz A. Taras D. Schäfer K. Beijer S. Bos N. A. Donus C.. (2010). Microbiota and SCFA in lean and overweight healthy subjects. Obes. (Silver Spring) 18, 190–195. doi: 10.1038/oby.2009.167 19498350

[B59] Shan Z. Y. Wang L. H. (2022). Guidelines for prevention and management of thyroid diseases during pregnancy and perinatal period. China J. Endocrinol. Metab. 38 (7). doi: 10.3760/cma.j.cn311282-20220416-00234

[B60] Sharma D. Shastri S. Sharma P. (2016). Intrauterine growth restriction: antenatal and postnatal aspects. Clin. Med. Insights Pediatr. 10, 67–83. doi: 10.4137/CMPed.S40070 27441006 PMC4946587

[B61] Sililas P. Huang L. Thonusin C. Luewan S. Chattipakorn N. Chattipakorn S.. (2021). Association between gut microbiota and development of gestational diabetes mellitus. Microorganisms 9 (8), 1686. doi: 10.3390/microorganisms9081686 34442765 PMC8400162

[B62] Stanislawski M. A. Dabelea D. Wagner B. D. Sontag M. K. Lozupone C. A. Eggesbø M. (2017). Pre-pregnancy weight, gestational weight gain, and the gut microbiota of mothers and their infants. Microbiome 5, 113. doi: 10.1186/s40168-017-0332-0 28870230 PMC5584478

[B63] Stavreus Evers A. (2012). Paracrine interactions of thyroid hormones and thyroid stimulation hormone in the female reproductive tract have an impact on female fertility. Front. Endocrinol. (Lausanne) 3, 50. doi: 10.3389/fendo.2012.00050 22649421 PMC3355884

[B64] Sun Y. Chen Q. Lin P. Xu R. He D. Ji W.. (2019). Characteristics of gut microbiota in patients with rheumatoid arthritis in Shanghai, China. Front. Cell Infect. Microbiol. 9, 369. doi: 10.3389/fcimb.2019.00369 31709198 PMC6819506

[B65] Tang L. Li P. Zhou H. Li L. (2021). A longitudinal study of thyroid markers during pregnancy and the risk of gestational diabetes mellitus and post-partum glucose metabolism. Diabetes Metab. Res. Rev. 37, e3441. doi: 10.1002/dmrr.3441 33486811 PMC8243952

[B66] Taylor P. N. Albrecht D. Scholz A. Gutierrez-Buey G. Lazarus J. H. Dayan C. M.. (2018). Global epidemiology of hyperthyroidism and hypothyroidism. Nat. Rev. Endocrinol. 14, 301–316. doi: 10.1038/nrendo.2018.18 29569622

[B67] Tomasello G. Tralongo P. Amoroso F. Damiani P. Sinagra E. Noto M.. (2015). Dysmicrobism, inflammatory bowel disease and thyroiditis: analysis of the literature. J. Biol. Regul. Homeost Agents 29, 265–272. 26122213

[B68] Topliss D. J. (2016). Clinical update in aspects of the management of autoimmune thyroid diseases. Endocrinol. Metab. (Seoul) 31, 493–499. doi: 10.3803/EnM.2016.31.4.493 28029020 PMC5195823

[B69] Tortosa F. (2011). [Subclinical thyroid dysfunction in pregnancy]. Endocrinol. Nutr. 58, 255–257. doi: 10.1016/j.endonu.2011.05.001 21722857

[B70] Tu X. Duan C. Lin B. Li K. Gao J. Yan H.. (2022). Characteristics of the gut microbiota in pregnant women with fetal growth restriction. BMC Pregnancy Childbirth 22, 297. doi: 10.1186/s12884-022-04635-w 35392848 PMC8991653

[B71] Unuane D. Velkeniers B. Anckaert E. Schiettecatte J. Tournaye H. Haentjens P.. (2013). Thyroglobulin autoantibodies: is there any added value in the detection of thyroid autoimmunity in women consulting for fertility treatment? Thyroid 23, 1022–1028. doi: 10.1089/thy.2012.0562 23405888 PMC3752510

[B72] van Dijk M. M. Vissenberg R. Fliers E. van der Post J. A. M. van der Hoorn M. P. de Weerd S.. (2022). Levothyroxine in euthyroid thyroid peroxidase antibody positive women with recurrent pregnancy loss (T4LIFE trial): a multicentre, randomised, double-blind, placebo-controlled, phase 3 trial. Lancet Diabetes Endocrinol. 10, 322–329. doi: 10.1016/S2213-8587(22)00045-6 35298917

[B73] Ventura R. E. Iizumi T. Battaglia T. Liu M. Perez-Perez G. I. Herbert J.. (2019). Gut microbiome of treatment-naïve MS patients of different ethnicities early in disease course. Sci. Rep. 9, 16396. doi: 10.1038/s41598-019-52894-z 31705027 PMC6841666

[B74] Wang H. Gao H. Chi H. Zeng L. Xiao W. Wang Y.. (2017). Effect of levothyroxine on miscarriage among women with normal thyroid function and thyroid autoimmunity undergoing *in vitro* fertilization and embryo transfer: A randomized clinical trial. Jama 318, 2190–2198. doi: 10.1001/jama.2017.18249 29234808

[B75] Wu Z. Cai Y. Xia Q. Liu T. Yang H. Wang F.. (2019). Hashimoto’s thyroiditis impairs embryo implantation by compromising endometrial morphology and receptivity markers in euthyroid mice. Reprod. Biol. Endocrinol. 17, 94. doi: 10.1186/s12958-019-0526-3 31729993 PMC6857235

[B76] Yoon K. Kim N. (2021). Roles of sex hormones and gender in the gut microbiota. J. Neurogastroenterol Motil. 27, 314–325. doi: 10.5056/jnm20208 33762473 PMC8266488

[B77] Zhao F. Feng J. Li J. Zhao L. Liu Y. Chen H.. (2018). Alterations of the gut microbiota in hashimoto’s thyroiditis patients. Thyroid 28, 175–186. doi: 10.1089/thy.2017.0395 29320965

[B78] Zhao H. Yuan L. Zhu D. Sun B. Du J. Wang J. (2022). Alterations and mechanism of gut microbiota in graves’ Disease and hashimoto’s thyroiditis. Pol. J. Microbiol. 71, 173–189. doi: 10.33073/pjm-2022-016 35675824 PMC9252144

[B79] Zhou P. Yao Q. Zhao Q. Yang L. Yu Y. Xie J.. (2022). IVF/ICSI outcomes of euthyroid infertile women with thyroid autoimmunity: does treatment with aspirin plus prednisone matter? BMC Pregnancy Childbirth 22, 263. doi: 10.1186/s12884-022-04532-2 35351031 PMC8966173

